# Effect of underactuated parallelogram shape-shifting for environmental adaptation movement of a three modular in-pipe robot

**DOI:** 10.3389/frobt.2023.1234835

**Published:** 2023-09-21

**Authors:** Atsushi Kakogawa, Shugen Ma

**Affiliations:** Department of Robotics, Ritsumeikan University, Kusatsu, Shiga, Japan

**Keywords:** underactuation, differential mechanism, environmental adaptation mechanism, in-pipe robot, crawler module

## Abstract

This paper presents an in-pipe robot with three underactuated parallelogram crawler modules, which can automatically shift its body shape when encountering obstacles. The shape-shifting movement is achieved by only a single actuator through a simple differential mechanism by only combining a pair of spur gears. It can lead to downsizing, cost reduction, and simplification of control for adaptation to obstacles. The parallelogram shape does not change the total belt circumference length, thus, a new mechanism to maintain the belt tension is not necessary. Moreover, the proposed crawler can form the anterior-posterior symmetric parallelogram relative to the moving direction, which generates high adaptability in both forward and backward directions. However, whether the locomotion or shape-shifting is driven depends on the gear ratio of the differential mechanism because their movements are only switched mechanically. Therefore, to clarify the requirements of the gear ratio for the passive adaptation, two outputs of each crawler mechanism (torques of the flippers and front pulley) are quasi-statically analyzed, and how the environmental and design parameters influence the robot performance are verified by real experiments. From the experiments, although the robot could not adapt to the stepped pipe in vertical section, it successfully shifted its crawler’s shape to parallelogram in horizontal section only with our simulated output ratio.

## 1 Introduction

Maintenance of pipelines is one of the recent consequential missions for urban society. For efficient replacement of aging pipelines, how to know damaged and deteriorated points is the key process. However, it is time-consuming, costly, and dangerous if the inspection is performed manually. To solve this problem, self-propelled in-pipe inspection robots are collecting attention. Previously, many types of the in-pipe robot have been already researched and developed (Hirose et al., 1999; Rome et al., 1999; Streich and Adria, 2004; Birkenhofer et al., 2005; Fjerdingen et al., 2009; Schempf et al., 2010; Dertien et al., 2011; 2014; Kakogawa and Ma, 2012; Nishimura et al., 2012; Debenest et al., 2014; Pfotzer et al., 2014; 2015; Inazawa et al., 2021; Liu et al., 2022).

Among them, articulated robot is one of the most adaptable strucre for narrow (less than 4 in inner diameter) and winding pipelines Dertien et al. (2011, 2014); Debenest et al. (2014); Fjerdingen et al. (2009), and we have been also tackling on this projects (Kakogawa and Ma, 2018; Kakogawa et al., 2019; 2022b; a). If the adaptive pipe size is not a big issue (more than 4 in inner diameter), in-pipe robots with three independent driving modules have been also reported as a reliable structure (Roh and Choi, 2005; Roh et al., 2009; Kim et al., 2010; Park et al., 2010; Kwon and Yi, 2012; Suryavanshi et al., 2020; Tugeumwolachot et al., 2022). They revealed that the robot can travel through straight pipes and also steer at three-dimensional T-branches by only adjusting the speed difference among the modules.

Most three modular in-pipe robot is equipped with three actuators for moving forward and backward, and one passive compliant contractile mechanism that presses the modules radially to the inner pipe wall. This contractile mechanism needs to keep expanding the crawler to the wall. Therefore, when encountering obstacles or entering into smaller diameter pipes, large propulsive force is necessary. As assistance of the shrinkage, the mechanism with additional actuators has been reported (Park et al., 2010). However, it causes a critical increase in the total size of the robot. The robot cannot be equipped with many actuators for switching movements or sensors for contact detection in limited space such as in-pipe. In those cases, a method that maximizes the functionality of the motor itself is required.

Therefore, we herein proposed a new in-pipe robot with three underactuated parallelogram crawler modules. The crawler module can shift its body shape to parallelogram to adapt to obstacles without any additional actuators and sensors. Two kinds of movement (traveling and shape-shifting) are switched by using a simple differential mechanism and utilizing the external forces from the obstacle. However, the two behaviors change depending on the gear ratio of the differential mechanism.

To design the gear ratio, we focuse on the required output ratio of the pulley and the flipper in the normal driving and parallelogram modes. And, they are analyzed based on quasi-statics. The influences of the roll angle of the robot, the initial resistance of the crawler, the slope angle of the pipe, and the frictional coefficient are also examined. Experiments in a tilted stepped pipe are conducted to verify the performance of the developed in-pipe robot depending on the different gear ratio. Our previous works have already proposed the same idea (Kakogawa and Ma, 2013; Kakogawa et al., 2014). However, this paper explains how to design the gear ratio of the differential mechanism more in detail and the experiments with more conditions are performed to verify the validity of the proposed robot.

Originally, a realistic model that accurately includes the three modules is necessary. However, in reality, the characteristics of the environment (especially the friction coefficient of the pipe’s inner wall) are indeterminate, and in the friction losses of the sliding parts of the three links and the individual differences of the motor characteristics cannot be ignored. Therefore, building a too accurate model would be almost meaningless. We performed a quasi-static analysis to obtain a rough estimate of the gear tooth ratio. It should be noted here that the torque required to overcome the obstacle was not obtained, but only the output ratio of the differential mechanism. In this case, if the output ratio is well designed and the torque generated by the actuator is sufficiently large, the operation can be switched without control depending on the external environment. This paper proposes a design method for this purpose.

## 2 Mechanical structure of the proposed in-pipe robot and the principle of the underactuated movement

### 2.1 Basic structure

**FIGURE 1:**
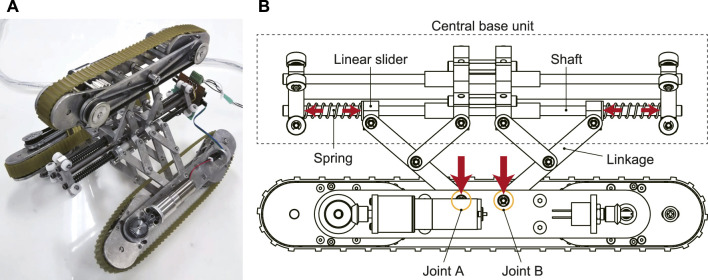
A mechanical model of our proposed in-pipe robot with underactuated parallelogram modules [**(A)**: overview picture, **(B)**: pantograph contractile mechanism].

Figure 1A shows the overview of the developed in-pipe robot. Its specifications are: *ϕ*136 mm—*ϕ*202 mm of the adaptive diameter, 235 mm of the axial length, 1.8 kg of the total weight, and 0.069 m/s of the moving speed. A geared motor (DCX16S GB kL 12 V and GPX19 C) with 231:1 reduction (Maxon group ag, Sachseln, Switzerland) was used for each crawler module. This robot is composed of a contractile mechanism and three crawler modules arranged radially at intervals of 120° with respect to the robot’s center axis.

A pantograph and a coil spring as shown in Figure 1B are installed for the expansion and contraction of the contractile mechanism. Two pantograph mechanisms and two coil springs are used for one crawler module. The slider that moves horizontally with respect to the robot center axis pushes the linkage of the pantograph mechanism with a spring, and the force generated by this pushes the joints A and B in the center of the crawler. The direction of the spring force is changed from the horizontal direction to the radial direction of the pipe.

### 2.2 Underactuated parallelogram crawler module

The contractile mechanism always presses each module against the inner wall of the pipe with a spring, thus it is difficult to shrink the robot body when an obstacle is encountered or the diameter of the pipe is reduced. Therefore, in this study, we developed a new shape-shifting mechanism called “underactuated parallelogram crawler” and installed it in three modules to address this problem. In general, active flipper arms and shape-shifting functions have often been deployed independently to solve such obstacle adaptation problems. However, in an environment that is restricted in space, such as in pipelines, adding actuators is a major barrier to downsizing. In this research, the power for moving forward and backward is utilized for shifting the crawler shape by an underactuated mechanism.

There are three reasons why a parallelogram shape is adopted for the crawler mechanism.

**FIGURE 2:**
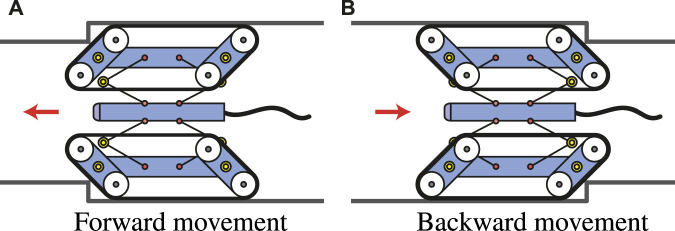
Similar mobility for anterior-posterior symmetric transformation [**(A)**: forward movement, **(B)**: backward movement].

1. When the crawler shape is shifted to a parallelogram, the mainframe of the crawler is lifted, and a force can be generated in the direction to shrink the contractile mechanism.
2. Even if the crawler shape is shifted to a parallelogram, the total belt circumference length is constant, thus, there is no need to add a new mechanism to maintain the belt tension.
3. Usually, in-pipe robots need to have the same function not only when moving forward but also when moving backward. A parallelogram can achieve anterior-posterior symmetric shape as illustrated in Figure 2. By using this symmetric shape-shifting function, even if it is difficult to pivot-turn, it can achieve the same transformation in the reverse direction by simply moving backward without changing the robot orientation.

**FIGURE 3:**
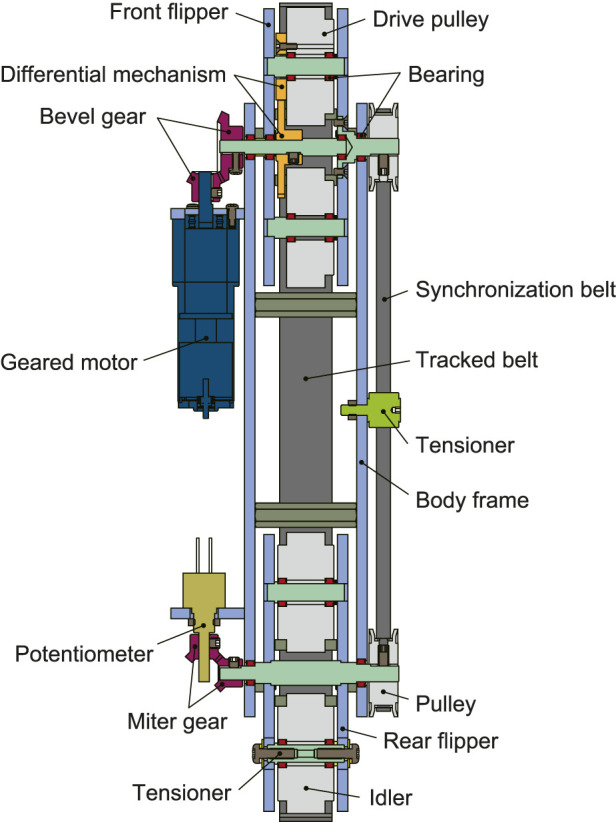
A CAD model of an underactuated parallelogram crawler module (cross-sectional view).

Figure 3 shows the cross-sectional view of a single underactuated parallelogram crawler. First, the motor is fixed to the outside of the crawler frame, and the power is transmitted to the differential mechanism in the front flipper via the bevel gear. With this differential mechanism, the power is transmitted to the drive pulley at the tip and the rotation of the flippers at the same time. The detailed principle is described later in the next subsection.

It is necessary to synchronize the rotation of the front and rear flippers to make the crawler parallelogram. In the real world, a parallel link mechanism is often used to synchronize the front and rear rotations, as seen in crank-bars of steam locomotives. However, during the normal driving in our case, the front and rear flippers are aligned in a straight line. For this reason, the flipper must be rotated from a singular posture, and the forward and backward movements are not uniquely determined (Ye et al., 2012). To solve this problem, the output of the front flipper is also transmitted to the rear flipper via a timing belt attached to the opposite side of the motor. As a result, the axes of the front and rear flippers are synchronized. Optionally, a potentiometer can be attached to the rear flipper to estimate the rotation angle of the flippers.

In conventional underactuated crawler robots, a reduction gear such as a planetary gear mechanism is often used to generate the differential movement (underactuated movement) (Lan et al., 2007; Kim et al., 2010; Quan and Ma, 2011; Wang et al., 2016). However, a large reduction gear cannot be used because the inner space of the pipe is limited. Therefore, in this study, a simple differential mechanism using a couple of spur gears is adopted (Li et al., 2010).

### 2.3 Pseudo multiple degrees of freedom by utilizing external forces

**FIGURE 4:**
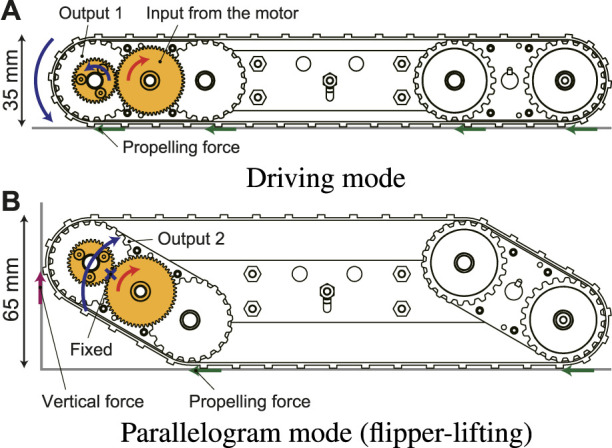
The two modes of the differential mechanism [**(A)**: driving mode, **(B)**: parallelogram mode].

Figure 4 shows the principle of the proposed underactuated parallelogram crawler as well as two modes of the normal driving and parallelogram. In the normal driving mode (Figure 4A), the front and rear flippers are horizontal because the crawler module is pressed against the inner wall of the pipe by the pantograph mechanism. In this state, the power is transmitted to the drive pulley at the front tip, and the crawler can move forward and backward.

On the other hand, when the crawler is in contact with an obstacle and the robot movement is intercepted, the drive pulley cannot be rotated around its own axis. As a result, it revolutes around the input gear and the crawler shape is shifted to a parallelogram as shown in Figure 4B.

In addition, since the pulley’s drive force is always generated even in contact with the obstacle, a vertically upward force is applied to the contact point of the crawler front portion. This force has an effect of assisting the rotation of the flipper. The flippers can be lifted without using a high reduction ratio thanks to this assistance. In other words, if the force acting on the crawler’s contact point can generate a moment in the direction of assisting the rotation of the flipper, the desired operation can be performed even at a low reduction ratio. Therefore, the number of gears can be greatly reduced compared with conventional underactuated crawler robots. This will lead to downsizing and lightweight.

Many existing crawler robots used in outdoor environments often improve obstacle-adaptability by preparing a tilt angle of the tracked-belt. However, since the size is limited in pipelines, a method of shifting the robot’s shape depending on the environment is more effective. Furthermore, when an unexpected external force is applied to the crawler during the travel, it can be mitigated using the rotation of the flipper. This can reduce the impact on the motor (Quan and Ma, 2011).

**FIGURE 5:**
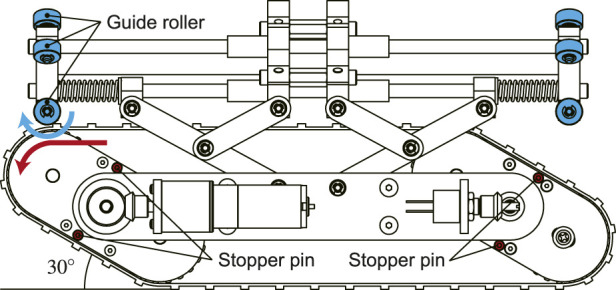
Stopper pins used to limit the rotational angle of the flippers and guide rollers to guarantee smooth motion of the tracked belt.

When the crawler contacts with the obstacle, the flippers rotate inward, and the shape is shifted to a parallelogram. However, if the resistance continues to be applied to the tracked-belt, the flippers keep rotating infinitely in the opposite direction to that of travel. Eventually, the robot cannot overcome the obstacle. To solve this, when the flipper is rotated to 30°, stopper pins are attached to prevent further rotation (Figure 5). If the motor continues to rotate in this state, the crawler can move forward by driving the tracked-belt while maintaining the parallelogram.

When the contractile mechanism shrinks while keeping a parallelogram shape of the crawler, the tracked-belt contacts with the central portion of the robot. Thus, a large resistance is generated that interrupts the robot movement. To reduce this resistance, guide rollers are attached to the front and rear tips of the robot as shown in Figure 5.

## 3 Quasi-static model

**FIGURE 6:**
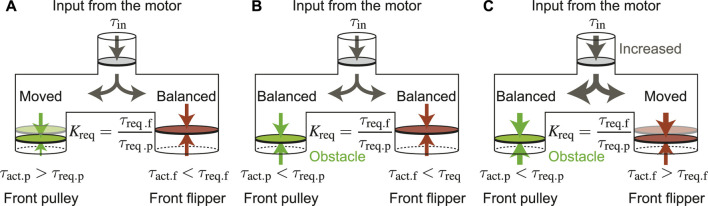
Branch pipe models for torque distribution: **(A)** Driving mode, **(B)** Between driving mode and parallelogram mode, **(C)** Parallelogram mode.

Since the underactuated mechanism divides one input into two outputs simultaneously, the distribution ratio of the two outputs greatly affects the performance. This differential phenomenon is often expressed using a branch tube as shown in Figure 6 (Lan et al., 2007). In normal driving mode, only the drive pulley rotates and the flipper must be stationary. In other words, the actual torque transmitted to the drive pulley *τ*_act.p_ must be larger than *τ*_req.p_. In the same manner, the actual torque transmitted to the flipper *τ*_act.f_ must be less than *τ*_req.f_. On the other hand, in parallelogram mode, the drive pulley should stop and the flipper must rotate. Therefore, *τ*_act.p_ must be smaller than *τ*_req.p_, and *τ*_act.f_ must be greater than *τ*_req.f_.

Here, the ratio between the torque required to rotate the drive pulley and the one required to lift the flipper and the actual output ratio are defined as follows, respectively.

$$K_{\text{req}}=\tau_{\text{req.f}}/\tau_{\text{req.p}}$$ (1)

$$K_{\text{act}}=\tau_{\text{act.f}}/\tau_{\text{act.p}}$$ (2)

in the driving mode, both of the following two conditions should be satisfied.

$$\tau_{\text{act.f}}<\tau_{\text{req.f}}$$ (3)

$$\tau_{\text{act.p}}>\tau_{\text{req.p}}$$ (4)

in the parallelogram mode, the following two conditions should be satisfied in a similar way.

$$\tau_{\text{act.f}}>\tau_{\text{req.f}}$$ (5)

$$\tau_{\text{act.p}}<\tau_{\text{req.p}}$$ (6)

therefore, *K*_act_ should meet the following conditions.

$$\left\{\begin{array}{cc} K_{\text{act}}<K_{\text{req}}\; & \left(Normal\;driving\;mode\right)\\ K_{\text{act}}>K_{\text{req}}\; & \left(Parallelogram\;mode\right) \end{array}\right.$$ (7)

**FIGURE 7:**
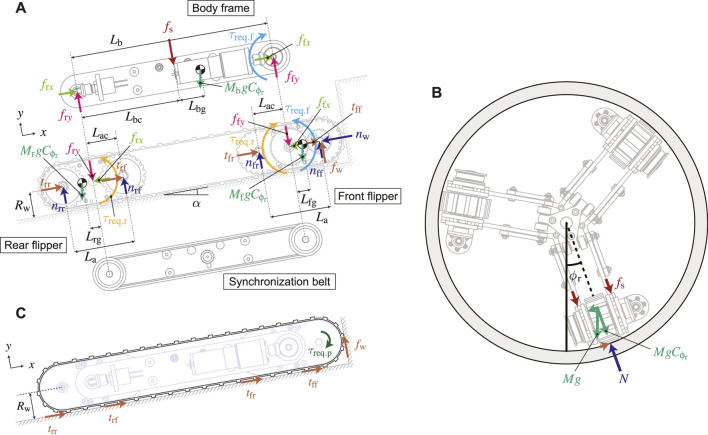
Possible external forces acting on the underactuated parallelogram crawler [**(A)**: forces of each rigid body, **(B)**: forces of the tracked belt, **(C)**: forces in the cross-sectional view].

Since *τ*_req.p_ and *τ*_req.f_ change depending on the environment the crawler encounters, these values are derived based on static models as shown in Figure 7A–C, respectively.

The static model is simplified by dividing the crawler into three rigid bodies: a front flipper, a rear flipper, and a body frame. To generalize, the crawler moves on the inner wall of the pipe tilted by *α*, and the front flipper is in contact with the step. The rotation axes of the front and rear flippers are at the respective centers and are fixed to both ends of the main body frame. In addition, since the axes of these flippers are synchronized by the timing belt, the torque required to rotate the rear flipper *τ*_req.r_ interact with the front flipper as inner torque. The motor input torque *τ*_req.f_ is applied to the front flipper, and its reaction is applied to the body frame.

Since the three crawler modules are arranged radially as shown in Figure 7B, the direction of gravity applied to the modules changes depending on the roll-posture of the robot. Therefore, each gravitational force acting in the radial direction of the pipe is calculated by $M_{\text{f}}gC_{\phi_{\text{r}}}$ , $M_{\text{b}}gC_{\phi_{\text{r}}}$ , and $M_{\text{r}}gC_{\phi_{\text{r}}}$ , respectively.

### 3.1 Torque for lifting the flippers

From Figure 7A, the torque required to lift the flipper can be obtained from the balance of external forces and moments acting on the three rigid bodies (front flipper, rear flipper, and body frame). This is because the required torque means a boundary value to break down the force balance. Considering the balance of forces in the *x* and *y*-axes and the moment around the rotation axis of the flipper, the static balance equations of the front flipper can be obtained by

$$t_{\text{ff}}+t_{\text{fr}}-f_{\text{f}x}-n_{\text{w}}-M_{\text{f}}gC_{\phi_{\text{r}}}S_{\alpha }=0$$ (8)

$$N_{\text{ff}}+n_{\text{fr}}-f_{\text{f}y}+f_{\text{w}}-M_{\text{f}}gC_{\phi_{\text{r}}}C_{\alpha }=0$$ (9)

$$\begin{array}{ll} & t_{\text{ff}}R_{\text{w}}+t_{\text{fr}}R_{\text{w}}+n_{\text{ff}}L_{\text{ac}}-n_{\text{fr}}L_{\text{ac}}+f_{\text{w}}L_{\text{ac}}\\ & \;\;+\tau_{\text{req.f}}-\tau_{\text{req.r}}-M_{\text{f}}gC_{\phi_{\text{r}}}L_{\text{fg}}C_{\alpha }=0 \end{array}$$ (10)

in the same way, the static balance equations of the body frame can be derived by

$$f_{\text{f}x}+f_{\text{r}x}-M_{\text{b}}gC_{\phi_{\text{r}}}S_{\alpha }=0$$ (11)

$$f_{\text{f}y}+f_{\text{r}y}-M_{\text{b}}gC_{\phi_{\text{r}}}C_{\alpha }-f_{\text{s}}=0$$ (12)

$$f_{\text{f}y}L_{\text{bc}}-f_{\text{r}y}L_{\text{bc}}-\tau_{\text{req.f}}-M_{\text{b}}gC_{\phi_{\text{r}}}L_{\text{bg}}C_{\alpha }=0$$ (13)

the static balance equations of the rear flipper are obtained by

$$t_{\text{rf}}+t_{\text{rr}}-f_{\text{r}x}-M_{\text{r}}gC_{\phi_{\text{r}}}S_{\alpha }=0$$ (14)

$$n_{\text{rf}}+n_{\text{rr}}-f_{\text{r}y}-M_{\text{r}}gC_{\phi_{\text{r}}}C_{\alpha }=0$$ (15)

$$t_{\text{rf}}R_{\text{w}}+t_{\text{rr}}R_{\text{w}}+n_{\text{rf}}L_{\text{ac}}-n_{\text{rr}}L_{\text{ac}}+\tau_{\text{req.r}}-M_{\text{r}}gC_{\phi_{\text{r}}}L_{\text{rg}}C_{\alpha }=0$$ (16)

They are basic equations for force and moment balance, thus, the applied external forces vary depending on the situation where the crawler is placed. Different torque conditions in two situations, the normal driving mode and the parallelogram mode, are obtained, respectively.

#### 3.1.1 Torque for lifting the flippers in the normal driving mode

In the normal driving mode, the tip of the crawler does not touch the obstacle. Therefore, *f*_w_ = *n*_w_ = 0 is obtained. Assuming that the flipper is slightly raised, *t*_ff_ = *t*_rf_ = *n*_ff_ = *n*_rf_ = 0. Substituting them into the above Eqs 8–10, the equations of equilibrium for the front flipper can be derived below:

$$t_{\text{fr}}-f_{\text{f}x}-M_{\text{f}}gC_{\phi_{\text{r}}}S_{\alpha }=0$$ (17)

$$n_{\text{fr}}-f_{\text{f}y}-M_{\text{f}}gC_{\phi_{\text{r}}}C_{\alpha }=0$$ (18)

$$t_{\text{fr}}R_{\text{w}}-n_{\text{fr}}L_{\text{ac}}+\tau_{\text{req.f}}-\tau_{\text{req.r}}-M_{\text{f}}gC_{\phi_{\text{r}}}L_{\text{fg}}C_{\alpha }=0$$ (19)

in a similar way, substituting the above conditions into Eqs 14–16, the equations of equilibrium for the rear flipper can be obtained.

$$t_{\text{rr}}-f_{\text{r}x}-M_{\text{r}}gC_{\phi_{\text{r}}}S_{\alpha }=0$$ (20)

$$n_{\text{rr}}-f_{\text{r}y}-M_{\text{r}}gC_{\phi_{\text{r}}}C_{\alpha }=0$$ (21)

$$t_{\text{rr}}R_{\text{w}}-n_{\text{rr}}L_{\text{ac}}+\tau_{\text{req.r}}-M_{\text{r}}gC_{\phi_{\text{r}}}L_{\text{rg}}C_{\alpha }=0$$ (22)

the equations of equilibrium for the body frame are the same as Eqs 11–13.

From Eqs 11, 17, 20, the sum of the tangential force of the crawler is

$$t_{\text{fr}}+t_{\text{rr}}=MgC_{\phi_{\text{r}}}S_{\alpha }$$ (23)

from Eqs 12, 18, 21, the sum of the normal forces is

$$n_{\text{fr}}+n_{\text{rr}}=MgC_{\phi_{\text{r}}}C_{\alpha }+f_{\text{s}}$$ (24)

Now, substituting Eqs 23, 24 into the sum of Eqs 19, 22, the torque to lift both front and rear flippers in the normal driving mode *τ*_req.f_ can be calculated as follows:

$$\begin{array}{ll} \tau_{\text{req.f}} & =L_{\text{ac}}\left(MgC_{\phi_{\text{r}}}C_{\alpha }+f_{\text{s}}\right)-MgC_{\phi_{\text{r}}}R_{\text{w}}S_{\alpha }\\ & \;+\left(M_{\text{f}}L_{\text{fg}}-M_{\text{r}}L_{\text{rg}}\right)gC_{\phi_{\text{r}}}C_{\alpha }+\tau_{\text{i}}. \end{array}$$ (25)

The first term depends on the gravitational force and the spring force of the contractile mechanism, meaning that these forces need to be overcome to lift the flippers. The second term depends on the inclination angle of the inner wall of the pipe *α*. The larger this angle is, the smaller the torque required to lift the flipper, thus, the sign is negative. The third term depends on the position of the center of gravity of the front and rear flippers. The farther the position of the center of gravity is from the rotational axis of the arm, the more susceptible it is to that effect. In an actual robot, there is an initial resistance that is generated by the mechanical frictions such as the forces applied to the bearings and shafts due to the tension of the belt, and the inertia, etc., which influence each other in a complicated manner. Therefore, this initial resistance is collectively defined as *τ*_i_ here.

#### 3.1.2 Torque for lifting the flippers in the parallelogram mode

In the parallelogram mode, it is assumed that the flippers are slightly lifted, thus, *t*_ff_ = *t*_rf_ = *n*_ff_ = *n*_rf_ = 0 can be obtained. However, the tip of the crawler is in contact with the obstacle. Therefore, *f*_w_ ≠ *n*_w_ ≠ 0, and the equations for the force-moment balance of the front flipper are given below:

$$t_{\text{fr}}-f_{\text{f}x}-n_{\text{w}}-M_{\text{f}}gC_{\phi_{\text{r}}}S_{\alpha }=0$$ (26)

$$n_{\text{fr}}-f_{\text{f}y}-f_{\text{w}}-M_{\text{f}}gC_{\phi_{\text{r}}}C_{\alpha }=0$$ (27)

$$t_{\text{fr}}R_{\text{w}}-n_{\text{fr}}L_{\text{ac}}+f_{\text{w}}L_{\text{ac}}+\tau_{\text{req.f}}-\tau_{\text{req.r}}-M_{\text{f}}gC_{\phi_{\text{r}}}L_{\text{fg}}C_{\alpha }=0$$ (28)

The equations of equillibrium for the rear flipper are the same as Eqs 20–22 as well as that for the body frame are the same as Eqs 11–13. From Eqs 11, 20, 26, the sum of the tangential force of the crawler is derived by

$$t_{\text{fr}}+t_{\text{rr}}=MgC_{\phi_{\text{r}}}S_{\alpha }+n_{\text{w}}$$ (29)

from Eqs 12, 21, 27, the sum of the normal forces applying on the crawler is

$$n_{\text{fr}}+n_{\text{rr}}=MgC_{\phi_{\text{r}}}C_{\alpha }+f_{\text{s}}-f_{\text{w}}$$ (30)

When the crawler contacts an obstacle and cannot move forward further, the tracked-belt slips. Since sliding friction force generates at this moment, the following relationship can be derived using the sliding friction coefficient *μ*_s_:

$$t_{\text{fr}}=\mu_{\text{s}}n_{\text{fr}}$$ (31)

$$t_{\text{rr}}=\mu_{\text{s}}n_{\text{rr}}$$ (32)

$$f_{\text{w}}=\mu_{\text{s}}n_{\text{w}}$$ (33)

substituting Eqs 29, 30, 33 into the sum of Eqs 22, 28, *τ*_req.f_ can be calculated by

$$\begin{array}{ll} \tau_{\text{req.f}} & =L_{\text{ac}}\left(MgC_{\phi_{\text{r}}}C_{\alpha }+f_{\text{s}}\right)-MgC_{\phi_{\text{r}}}R_{\text{w}}S_{\alpha }+\left(M_{\text{f}}L_{\text{fg}}\right.\\ & \;+\left.M_{\text{r}}L_{\text{rg}}\right)gC_{\phi_{\text{r}}}C_{\alpha }-n_{\text{w}}\left\{2\mu_{\text{s}}L_{\text{a}}+R_{\text{w}}\left(1+\mu_{\text{s}}\right)\right\}+\tau_{\text{i}} \end{array}$$ (34)

from Eqs 29–33, the normal force that disturbs the crawler movement *n*_w_ is obtained by

$$n_{\text{w}}=\frac{\mu_{\text{s}}\left(MgC_{\phi_{\text{r}}}C_{\alpha }+f_{\text{s}}\right)-MgC_{\phi_{\text{r}}}S_{\alpha }}{\mu_{\text{s}}^{2}+1}$$ (35)

In a similar way as Eq. 25, the first term in Eq. 34 depends on the gravitational force and the spring force of the contractile mechanism. The second term depends on the inclination angle of the inner wall of the pipe *α*, and the larger this angle is, the torque necessary to lift the flippers reduces. The third term also depends on the position of the center of gravity of the front and rear flippers, similar to Eq. 25. The fourth term depends on the upward force applying between the crawler and the obstacle *f*_w_, and this force assists the rotation of the flippers. *τ*_i_ is also defined as the initial resistance.

### 3.2 Torque for rotating the pulley of the tracked-belt

The torque for rotating the pulley at the tip *τ*_req.p_ is generated from the torque for rotating the front flipper *τ*_req.f_ transmitted via the spur gear. It can be assumed that this torque is balanced with the forces applying along the crawler’s belt as shown in Figure 7C. Considering the initial resistance *τ*_i_ in the same manner, *τ*_req.p_ is given by

$$\tau_{\text{req.p}}=R_{\text{w}}\left(t_{\text{ff}}+t_{\text{fr}}+t_{\text{rf}}+t_{\text{rr}}+f_{\text{w}}\right)+\tau_{\text{i}}$$ (36)

as mentioned in the section of the torques for lifting the flipper in the normal driving mode and the parallelogram mode, the torque for rotating the pulley at the tip *τ*_req.p_ varies between two modes. Therefore, they are derived in each mode as follows.

#### 3.2.1 Torque for rotating the pulley in the normal driving mode

In the normal driving mode, *f*_w_ = *n*_w_ = 0 because the tip of the crawler does not contact the obstacle. Therefore, *τ*_req.p_ can be obtained by removing *f*_w_ from Eq. 36.

$$\tau_{\text{req.p}}=R_{\text{w}}\left(t_{\text{ff}}+t_{\text{fr}}+t_{\text{rf}}+t_{\text{rr}}\right)+\tau_{\text{i}}$$ (37)

eliminating *n*_w_ from the sum of Eqs 8, 11, 14 and substituting them into Eq. 37, *τ*_req.p_ can be obtained by

$$\tau_{\text{req.p}}=R_{\text{w}}MgC_{\phi_{\text{r}}}S_{\alpha }+\tau_{\text{i}}$$ (38)

#### 3.2.2 Torque for rotating the pulley in the parallelogram mode

On the other hand, in the parallelogram mode, the tip of the crawler contacts the obstacle, and the front and rear flippers are lifted. Therefore, *f*_w_ ≠ *n*_w_ ≠ 0 and *t*_ff_ = *t*_rf_ = 0. As a result, *τ*_req.p_ is given by

$$\tau_{\text{req.p}}=R_{\text{w}}\left(t_{\text{fr}}+t_{\text{rr}}+f_{\text{w}}\right)+\tau_{\text{i}}$$ (39)

substituting Eqs 29, 33 into Eq. 39, the torque for rotating the pulley in the parallelogram mode *τ*_req.p_ can be derived by

$$\tau_{\text{req.p}}=R_{\text{w}}\left\{MgC_{\phi_{\text{r}}}S_{\alpha }+\left(\mu_{\text{s}}+1\right)n_{\text{w}}\right\}+\tau_{\text{i}}$$ (40)

*n*_w_ can be obtained from Eq. 35.

## 4 Gear ratio design

*τ*_req.p_, *τ*_req.f_ obtained by the static analysis, and their output ratio *K* are given by Eqs 25, 38 in the normal driving mode and Eq. 34, 40 in the parallelogram mode. The parameters of each rigid body used in the analysis are: *M*_f_ [kg] = 0.13, *M*_b_ [kg] = 0.20, *M*_r_ [kg] = 0.12, *g* [m/s^2^] = 9.8, *R*_w_ [mm] = 20, *L*_a_ [mm] = 46, and *L*_b_ [mm] = 150. Since the rear flipper of the crawler and the mainframe have a symmetrical structure, *L*_bg_ = *L*_rg_ = 0 is assumed. Also, the center of gravity of the front flipper is moved only by the gear of the tip pulley, and this influence is small, thus, *L*_fg_ = 0. For the sliding friction coefficient *μ*_s_, 0.4 of the general polyurethane belt used for crawlers and vinyl chloride used for pipes is used.

For the initial resistance *τ*_i_, the value when the real crawler was idling with no load, which is calculated by the following equation using the motor current *i* at no-load rotation, torque constant *K*_*τ*_, and reduction ratio *K*_ratio_.

$$\tau_{\text{i}}=K_{\text{ratio}}K_{\tau }i$$ (41)

the current value is the measured value *i* = 0.054 A, the torque constant is *K*_*τ*_ = 8.83 Nmm/A from the motor specifications, and the reduction ratio is the actual value *K*_ratio_ = 231 is used. Based on them, *τ*_i_ = 106.4 Nmm is obtained.

### 4.1 Influence of the expanding force of the contractile mechanism *f*_s_ and determination of the spring stiffness

**FIGURE 8:**
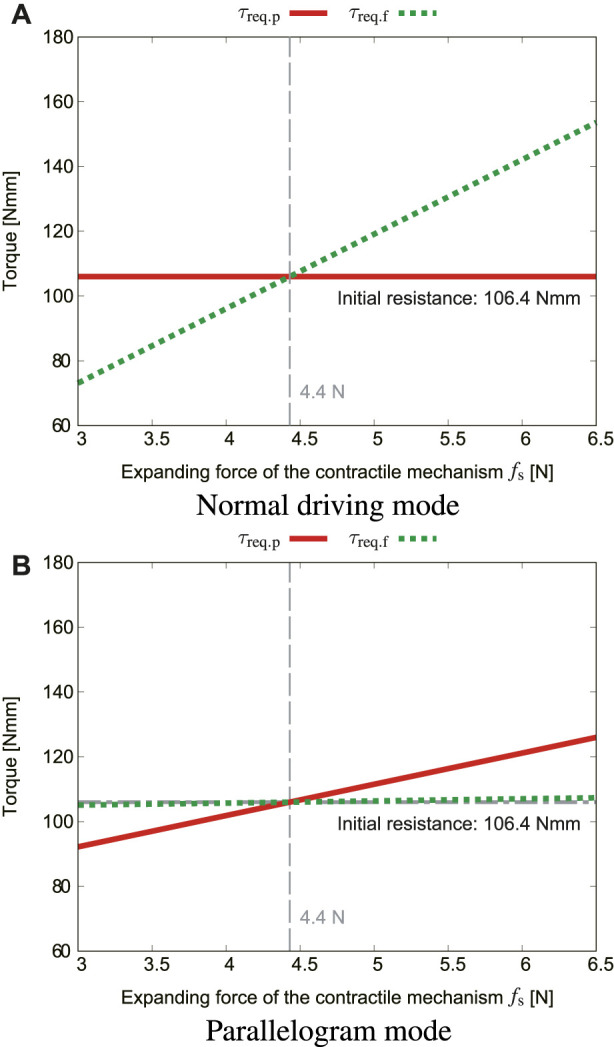
Effects of the expanding force *f*_s_, where *μ*_s_ = 0.4, *α* = 0, *ϕ*_r_ = 180° [**(A)**: normal driving mode, **(B)**: parallelogram mode].

When the crawler module is located on the upper inner wall of the pipe, the contractile mechanism needs to press the crawler with a larger force against gravity. Therefore, the influence of the expanding pressing force *f*_s_ of the contractile mechanism is calculated under the conditions of *α* = 0 and *ϕ*_r_ = 180°. Figure 8A, B show the effects of *f*_s_ in the normal driving mode and that of *f*_s_ in the parallelogram mode, respectively. When *f*_s_ is 4.4 N or less, *τ*_req.p_ and *τ*_req.f_ are both less than *τ*_i_. This means that the crawler moves away from the inner surface of the pipe, in other words, *f*_s_ should be at least 4.4 N.

The expansion force of the contractile mechanism is influenced by the spring force *s*_p_. The relationship between those two forces can be obtained by the following equation with the tangent function, referring to the literature (Kwon and Yi, 2012).

$$f_{\text{s}}=2s_{\text{p}}\tan\psi$$ (42)

in our case, the actual natural length of the spring is 55 mm, and it shrinks to 53 mm when the robot enters the straight pipe. Therefore, the spring force is given by *s*_p_ = *K*_s_(55 − 53) (*k* denotes the spring stiffness). In the straight pipe, *ψ* = 70°, and *K*_s_ = 0.5 N/mm was selected because *f*_s_ is approximately 5.5 N, which satisfies the condition of 4.4 N or higher.

### 4.2 Influence of the robot roll angle *ϕ*_r_

**FIGURE 9:**
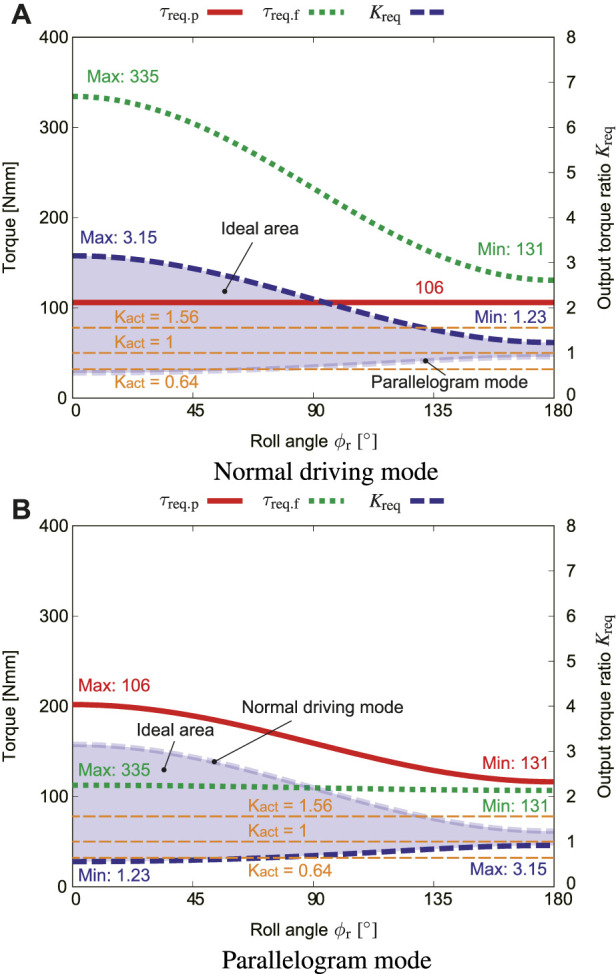
Effect of the roll angle *ϕ*_r_, where *μ*_s_ = 0.4, *f*_s_ = 5.5 N, *α* = 0 [**(A)**: normal driving mode, **(B)**: parallelogram mode].

Figure 9A shows how the roll angle of the robot *ϕ*_r_ influences *τ*_req.p_, *τ*_req.f_, and output ratio *K*_req_ in the normal driving mode. For *f*_s_, the above-mentioned value of 5.5 N was substituted. In a similar way, Figure 9B shows how *ϕ*_r_ influences *τ*_req.p_, *τ*_req.f_, and output ratio *K*_req_ in the parallelogram mode. In order to make the robot perform the ideal motion, the actual output ratio *K*_act_ should be smaller than *K*_req_ in the normal driving mode but greater than that in the parallelogram mode according to Eq. 7. Combining these two graphs, the output ratio should be within the range of 0.98 < *K*_act_ < 1.23 to fit with all roll angles range *ϕ*_r_. When *K*_act_ < 0.98, even if the robot contacts with an obstacle, the flipper cannot be lifted and keeps slipping. When 1.36 > *K*_act_, the flipper is lifted even in the normal driving mode.

### 4.3 Influence of the initial resistance *τ*_i_

**FIGURE 10:**
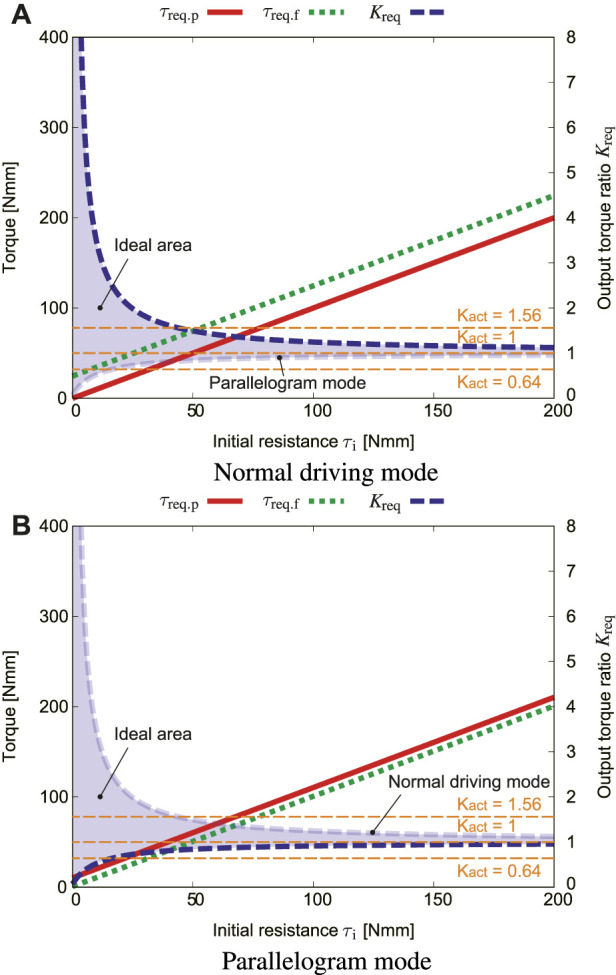
Effect of the initial friction *τ*_i_, where *μ*_s_ = 0.4, *f*_s_ = 5.5 N, *α* = 0, *ϕ*_r_ = 180° [**(A)**: normal driving mode, **(B)**: parallelogram mode].

Figure 10A plots how the initial resistance *τ*_i_ influences *τ*_req.p_, *τ*_req.f_, and output ratio *K*_req_ in the normal driving mode. From Figure 9, the rolling angle at the most severe condition (*K*_req_ range is the narrowest) *ϕ*_r_ = 180° was used. Figure 10B shows how *τ*_i_ influences *τ*_req.p_, *τ*_req.f_, and output ratio *K*_req_ in the parallelogram mode.

The results revealed that the initial resistance *τ*_i_ should be small to increase the range of *K*_req_. On the other hand, as the initial resistance increases, the values of *K* both approach one, thus the range of *K*_req_ is very narrow. If structures that cause a large initial resistance are installed, the output ratio must be set to one, which leads to a decrease in the option of the reduction ratio.

### 4.4 Influence of the pipe slope angle *α*

**FIGURE 11:**
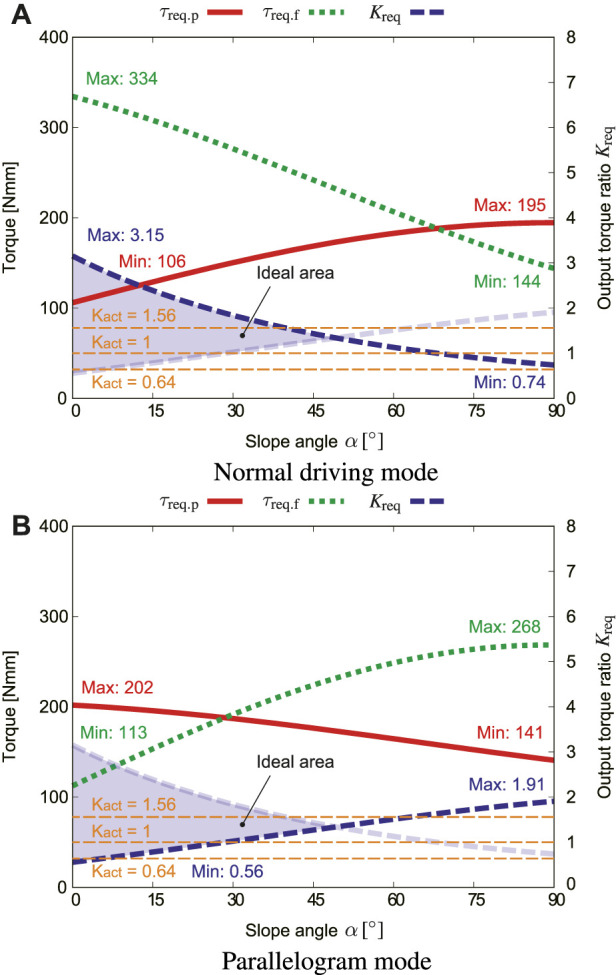
Effect of the slope angle *α*, where *μ*_s_ = 0.4, *f*_s_ = 5.5 N, *ϕ*_r_ = 0 [**(A)**: normal driving mode, **(B)**: parallelogram mode].

Figure 11A shows how the pipe slope angle *α* influences *τ*_req.p_, *τ*_req.f_, and output ratio *K*_req_ in the normal driving mode. In the normal driving mode, *τ*_req.p_ is maximum where *α* = 90°, which corresponds to the vertical pipe. The torque *τ*_req.f_ for lifting the flipper is inversely proportional to *α*. This means that as *α* increases, gravity helps the flippers rotate.

Figure 11B shows how *α* influences *τ*_req.p_, *τ*_req.f_, and output ratio *K*_req_ in the parallelogram mode. Since Eq. 34 is a function that includes both sin *α* and cos *α*, *τ*_req.f_ is maximum near *α* = 60°. The two *K*_req_ curves intersect at about *α* = 47°. This means that if *α* exceeds 47°, the flipper will be lifted even in the normal driving mode. However, in reality, when the flipper is lifted, the contractile mechanism is also shrunk, then *f*_s_ increases, and the flipper stops rotating and restarts the movement.

The ideal range of *K*_act_ gradually narrows as *α* increases. In this study, the actual output ratio is set to three variations: *K*_act_ = 1.56, *K*_act_ = 1, and *K*_act_ = 0.64 to fit with as wide range of *α* as possible. This is the value where the gear teeth number ratio is 56 : 36, 46 : 46, and 36 : 56, respectively. The output ratio *K*_act_ = 1.56 exceeds *K*_req_ at around *ϕ*_r_ = 135° as illustrated in Figure 9. This means that the flipper may be rotated when the crawler is flipped and located at the top inner surface of the pipe. However, this will not influence the forward and backward movement of the robot, because it can move even with the parallelogram shape.

### 4.5 Influence of the sliding frictional coefficient *μ*_s_

**FIGURE 12:**
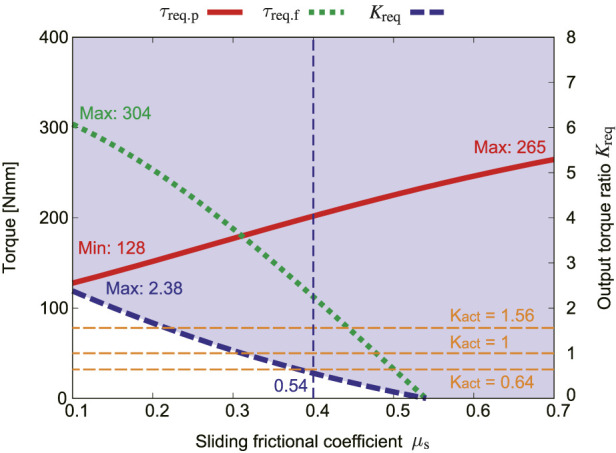
Effect of the sliding frictional coefficient *μ*_s_ in the parallelogram mode, where *f*_s_ = 5.5 N, *ϕ*_r_ = 0, *α* = 0.

The sliding friction coefficient *μ*_s_ is not taken into account in the normal driving mode. Therefore, only the influence of *μ*_s_ in the parallelogram mode is described here. Figure 12 shows how *μ*_s_ influences *τ*_req.p_, *τ*_req.f_, and output ratio *K*_req_ in the parallelogram mode. The torque for lifting the flipper *τ*_req.f_ has a negative value when *μ*_s_ exceeds around 0.8. This means that if *μ*_s_ is sufficient, the torque for rotating the flipper is not needed, and the crawler can shift its shape by only using the pulley rotation. When the sliding friction coefficient *μ*_s_ is less than around 0.2, *K*_act_ needs to be larger than 1.56, and the flipper cannot be lifted at this state because the crawler slips. The larger the sliding friction coefficient *μ*_s_, the wider the ideal range of *K*_act_. With this condition, the crawler can perform the adaptive movement to the environment even with a small output ratio.

The rated torque of the geared motor used for the proposed robot is 1,230 Nmm. The maximum value of the torque required to rotate the flipper *τ*_req.f_ is 335 Nmm according to Figures 9, 11. This satisfies the requirement. However, considering further safety factor, the torque of the motor is amplified twice by the bevel gear.

## 5 Experiments

### 5.1 Experimental setup

**FIGURE 13:**
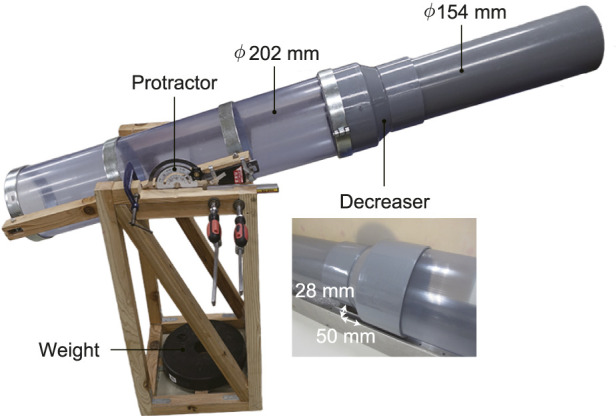
Experimental setup of a straight stepped pipe with a slope adjustment mechanism.

Adaptability of the proposed robot with underactuated parallelogaram modules was tested in a straight stepped pipe as shown in Figure 13. A standard constricted pipe called decreser was connected with two straight pipes with different diamiter; 202 mm (8 in) and 154 mm (6 in). The influence of the pipe slope angle *α* can be also examined by using a slope adjustment mechanism.

**FIGURE 14:**
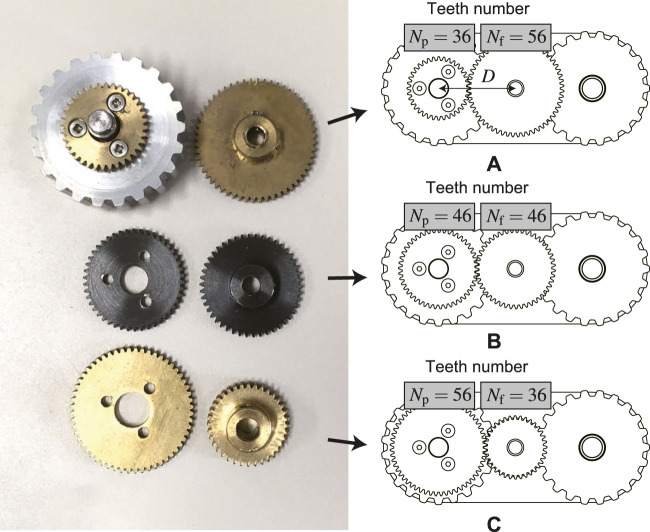
Three types of gear ratio: **(A)** Speed multiplication *K*_act_ = 1.56, **(B)** No speed change *K*_act_ = 1, **(C)** Speed reduction *K*_act_ = 0.64.

To verify the performance with different gear ratio (output ratio *K*_act_), three types of teeth number conbination: *K*_act_ = 1.56, *K*_act_ = 1, and *K*_act_ = 0.64 are prepared as depicted in Figure 14.

Each teeth number of two spur gears needs to satisfy the following condition:

$$N_{\text{f}}+N_{\text{p}}=\frac{2D}{N_{mod}}$$ (43)

where *N*_f_, *N*_p_, *D*, and *N*_mod_ denote the teeth number of the input (flipper) and output (pulley) gears, distance between two gear centers, and the gear module (the ratio of the reference diameter of the gear divided by the number of teeth), respectively. In our case, *D* = 23 mm and *N*_mod_ = 0.5, thus, the sum of *N*_f_ and *N*_p_ should keep 92.

Three motor drivers combining an ESCON 50/5 (Maxon group ag, Sachseln, Switzerland) and a microcontroller were fabricated for smooth experimentation, and control commands were given to each of them to keep the rotation speed at 40 rpm via CAN communication. The control commands were given via a graphical user interface (GUI) created with Visual Studio C#.

### 5.2 Experimental results

The experiment was conducted three times each with the following five combinations: *α* = 0 & *ϕ*_r_ = 0, *α* = 0 & *ϕ*_r_ = 180°, *α* = 45° & *ϕ*_r_ = 0, *α* = 45° & *ϕ*_r_ = 180°, and *α* = 90°. Upper table in Table 1 lists the success number in each trial where *K*_s_ = 0.5 N/mm.

From the experiment, it was found that the robot had the best step-adaptability when the output ratio was *K*_act_ = 0.64. It indicates that it is better to distribute the torque to the pulley than to the flipper. This result differs from the analysis in the previous section. However, the crawler module was able to deform into a parallelogram shape upon contacting the step in all results. The front half of the robot then slid to a stop as it entered the next narrow pipe (6 in). In all cases, the reason for the inability to travel to the narrow pipe was that the propulsive force of the crawler module was not sufficient to shurink the contractile mechanism.

**TABLE 1:** Experimental results where *K*_s_ = 0.5 N/mm (upper) and *K*_s_ = 0.3 N/mm (lower).

*K*_act_	*α* = 0 & *ϕ*_r_ = 0	*α* = 0 & *ϕ*_r_ = 180°	*α* = 45° & *ϕ*_r_ = 0	*α* = 45° & *ϕ*_r_ = 180°	*α* = 90°
0.64	3/3	2/3	0/3	2/3	0/3
1	2/3	1/3	0/3	0/3	0/3
1.56	0/3	0/3	0/3	0/3	0/3

*K*_act_	*α* = 0 & *ϕ*_r_ = 0	*α* = 0 & *ϕ*_r_ = 180°	*α* = 45° & *ϕ*_r_ = 0	*α* = 45° & *ϕ*_r_ = 180°	*α* = 90°
0.64	2/3	3/3	1/3	2/3	0/3
1	0/3	0/3	0/3	0/3	0/3
1.56	2/3	0/3	0/3	0/3	0/3

On the other hand, experiments were also conducted for *K*_s_ = 0.3 N/mm, which had been excluded because the analysis resulted the difficulty of adaptation to the step. The results of the experiment are listed in the lower table of Table 1. Overall, the results with *K*_act_ = 0.64 tended to be better, and in all cases the crawler module was able to shift its shape into a parallelogram. However, the performance at *K*_act_ = 1.56 was slightly better than that in the experiments with *K*_s_ = 0.3 N/mm.

Overall, the results with *K*_act_ = 0.64 tended to be better, and in all cases the crawler module was able to shift its shape into a parallelogram. However, the performance at *K*_act_ = 1.56 was slightly better than that in the experiments with *K*_s_ = 0.3 N/mm.

Some problems were found as a result of the experiments in the stepped pipe. For example, even when the robot adapted from a large-diameter to a small-diameter pipe, it sometimes did not return to its initial pose after returning from a small-diameter to a large-diameter pipe. Pressing the three crawler modules against the wall by a spring force large enough for the mass of the central part of the robot could solve this problem. However, the weight of the central part of the robot must be drastically reduced and the motor power must be increased due to the increased spring force.

On the other hands, even if the robot did not return to the original pose, it can be solved by controlling only each crawler module individually. For example, if only the contractile mechanism of the lower module shrinks, only that module will shift to parallelogram shape when only the lower one is driven and the other two upper modules are not. After repeating this operation several times, the pose will return to its original state (straightened). This is a disadvantage of the underactuated mechanism, but it is also an advantage of requiring fewer motors.

## 6 Conclusion

This paper proposes an environmental adaptation in-pipe robot with underactuated parallelogram crawlers. Without using additional motors and sensors, each crawler module can shift its body shape to a parallelogram to adapt to obstacles. The shape-shifting movement is generated by a simple differential mechanism of a pair of spur gears. However, whether the crawler moves forward or shifts the body shape depends on the gear ratio of the differential mechanism. To design the gear ratio, the required output ratio of the pulley to rotate and the flipper to lift up in the normal driving and parallelogram modes was analyzed quasi-statically. The influences of the roll angle of the robot, the initial resistance of the crawler, the slope angle of the pipe, and the frictional coefficient were also simulated. To examine the adaptability performance depending on the gear ratio, experiments in a tilted stepped pipe with a developed in-pipe robot were performed.

The robot successfully shifted its crawler’s shape to parallelogram only with our simulated output ratio, which implies that our quasi-static analysis is valid. In addition to the shape-shifting, the propulsive force for the contraction of the contractile mechanism and friction coefficient of the tracked belt were also found to be important for the adaptation to the stepped pipe. However, in tilted pipe experiments, the robot could not overcome the step, while it could climb the straight section against the gravity. This is because the unexpected uneven posture of the contractile mechanism was generated. One possible solution could be changing the stiffness of the contractile mechanism, but the gear ratio should be also redesigned again to match with this. Expanding the distance between two pantographs at each crawler module may make the contractile mechanism stiff against the external force. This would also solve the uneven posture problem.

## Figures and Tables

**Table:** 

*t*_ff_	Tangential force of the front pulley
*t*_fr_	Tangential force of the second pulley
*t*_rf_	Tangential force of the third pulley
*t*_rr_	Tangential force of the aftermost pulley
*n*_ff_	Normal force of the front pulley
*n*_fr_	Normal force of the second pulley
*n*_rf_	Normal force of the third pulley
*n*_rr_	Normal force of the aftermost pulley
*N*	Sum of the normal forces (*N* = *n*_ff_ + *n*_fr_ + *n*_rf_ + *n*_rr_)
*f*_w_	Vertical force of the front pulley
*n*_w_	Normal force stopping the crawler’s motion
*f*_f*x*_	*x*-axial force interacting between the front flipper and body frame
*f*_f*y*_	*y*-axial force interacting between the front flipper and body frame
*f*_r*x*_	*x*-axial force interacting between the rear flipper and body frame
*f*_r*y*_	*y*-axial force interacting between the rear flipper and body frame
*f*_s_	Expanding force of the contractile mechanism
*M*_f_	Mass of the front flipper
*M*_b_	Mass of the body frame
*M*_r_	Mass of the rear flipper
*M*	Total mass of the crawler module (*M* = *M*_f_ + *M*_b_ + *M*_r_)
*τ*_req.p_	Torque of the front driving pulley required to move the crawler
*τ*_req.f_	Torque required to lift the front flipper
*τ*_req.r_	Torque required to lift the rear flipper
*τ*_in_	Input torque from the geared motor
*τ*_act.p_	Actual torque transmitted to the drive pulley
*τ*_act.f_	Actual torque transmitted to the front flipper
*τ*_i_	iInitial resistance torque that is generated by the mechanical frictions
*K*_req_	Ratio between *τ*_req.p_ and *τ*_req.f_ (*K*_req_ = *τ*_req.f_/*τ*_req.p_)
*K*_act_	Ratio between *τ*_act.p_ and *τ*_act.f_ (*K*_act_ = *τ*_act.f_/*τ*_act.p_)
*g*	Acceleration of gravity
*μ*_r_	Coefficient of rolling friction
*μ*_s_	Coefficient of sliding friction
*α*	Inclination angle of the pipe
*ϕ*_r_	Roll angle of the robot around the axis of the pipe
*S*_*θ*_ and *C*_*θ*_	Sine and cosine function (sin *θ* and cos *θ*)
*R*_w_	Radii of pulleys
*L*_a_	Distance between pulleys of the front and rear flippers
*L*_ac_	Half length of *L*_a_
*L*_fg_	Distance between COG and center of the front flipper
*L*_rg_	Distance between COG and center of the rear flipper
*L*_b_	Length of the body frame
*L*_bg_	Distance between COG and center of the body frame
*L*_bc_	Half length of *L*_b_

## Data Availability

The datasets presented in this article are not readily available because Raw data cannot be published and shared. Requests to access the datasets should be directed to kakogawa@fc.ritsumei.ac.jp.
